# Database Survey of Anti-Inflammatory Plants in South America: A Review

**DOI:** 10.3390/ijms12042692

**Published:** 2011-04-21

**Authors:** Gedson Rodrigues de Morais Lima, Camila de Albuquerque Montenegro, Cynthia Layse Ferreira de Almeida, Petrônio Filgueiras de Athayde-Filho, José Maria Barbosa-Filho, Leônia Maria Batista

**Affiliations:** Laboratory of Pharmaceutical Technology, Department of Pharmaceutical Sciences, Federal University of Paraiba, 58051-970, João Pessoa, PB, Brazil; E-Mails: gedson@ltf.ufpb.br (G.R.M.L.); camila_montenegro@ltf.ufpb.br (C.A.M.); cynthialayse@gmail.com (C.L.F.A.); athayde-filho@quimica.ufpb.br (P.F.A.-F.); jbarbosa@ltf.ufpb.br (J.M.B.-F.)

**Keywords:** anti-inflammatory activity, leukocytes, medicinal plants, natural products, South American, review

## Abstract

Inflammation is a complex event linked to tissue damage whether by bacteria, physical trauma, chemical, heat or any other phenomenon. This physiological response is coordinated largely by a variety of chemical mediators that are released from the epithelium, the immunocytes and nerves of the lamina propria. However, if the factor that triggers the inflammation persists, the inflammation can become relentless, leading to an intensification of the lesion. The present work is a literature survey of plant extracts from the South American continent that have been reported to show anti-inflammatory activity. This review refers to 63 bacterial families of which the following stood out: Asteraceae, Fabaceae, Euphorbiaceae, Apocynaceae and Celastraceae, with their countries, parts used, types of extract used, model bioassays, organisms tested and their activity.

## 1. Introduction

Inflammation is the response of body to injury and danger. It is the central communication network and regulatory process that senses and controls threat, damage, containment, and healing, which are all critical aspects in the maintenance of the integrity of an organism [1].

This process occurs as a defensive response, which induces profound physiological adaptions triggered in an attempt to limit tissue damage and remove the pathogenic insult. Such mechanisms involve a complex series of events including dilatation of arterioles, venules and capillaries with increased vascular permeability, exudation of fluids, including plasma proteins, and leukocyte migration into the inflammatory area [2].

In response to injury or infection, the specialized cells of the first line, leukocytes (neutrophils and eosinophils polymorphonuclear-PMNs) migrate to the damaged regions with the aim of neutralizing and eliminating these harmful stimuli [3]. The mechanism of inflammation is attributed, in part, to release of reactive oxygen species (ROS) from activated neutrophils and macrophages [4]. ROS propagate inflammation by stimulating release of cytokines, such as interleukin-1, tumor necrosis factor-α, and interferon-γ, which stimulate recruitment of additional neutrophils and macrophages. Thus free radicals are important mediators that provoke or sustain inflammatory processes and, consequently, their neutralization by antioxidants and radical scavengers can attenuate inflammation [5,6].

A complex network of mediators, including cytokines and lipids, produced by endothelial cells, epithelial cells and tissue infiltrating leukocytes, characterizes the early phases of inflammation [7].

The clinical features of inflammation were described some 2000 years ago listed as the cardinal signs of inflammation: rubor (redness), tumor (swelling), heat (hyperthermia) and pain [8].

The combined action of the molecules attracts and activates leukocytes to the reactive site, promotes angiogenesis and tissue remodeling [7]. If this sequence of steps is rigorously followed, the acute inflammation will resolve without causing excessive damage to tissue, returning to homeostasis [3].

However, there are several clinical conditions where inflammation becomes chronic with excessive production of macrophage-derived mediators may lead to collateral damage to normal cells, which results in diseases, including atherosclerosis, bowel disease, rheumatoid arthritis glomerulonephritis, and septic shock [9].

Therefore, the classical anti-inflammatory agents glucocorticoids and non-steroidal anti-inflammatory drugs (NSAIDs) can only alleviate symptoms without, however, altering the course of the disease [3].

The current anti-inflammatory therapy aims to control the cardinal signs of inflammation, antagonizing or blocking key pro-inflammatory mediators that are released at the beginning of an acute inflammatory response [3]. NSAIDs typically relieve inflammation and associated pain by inhibiting cyclooxygenase enzymes involved in the production of prostaglandins. These enzymes exist in two isoforms (COX-1 and COX-2) coded by distinct genes on different chromosomes [10]. Compounds that inhibit COX enzymes could therefore be considered to be potential anti-inflammatory drugs. However, many of the commonly used anti-inflammatory agents are becoming less acceptable due to serious adverse reactions such as gastric intolerance, bone marrow depression and water and salt retention, resulting from prolonged use [11].

Within this context, it is of fundamental importance to search for substances that can promote the resolution of inflammation, thus, homeostatic and modulatory, efficient and tolerated by the body [3].

Plants are an important source of biologically active natural products and are considered a promising avenue for the discovery of new drugs due to easy access and relatively low cost, since they naturally grow in relative abundance [12,13]. The development of standardized herbal medicines with proven efficacy and safety of use is an important source for increasing the access of people to medicines and to offer new therapeutic options [14].

So, can cite examples of plants with scientifically proven anti-inflammatory activity: *Annona muricata*, *Glycine max*, *Orthosiphon stamineus*, *Caulerpa racemosa* and *Oenothera speciosa* used in folk medicine [15–19].

Therefore, extracts or isolated compounds from natural products seems to be a promising strategy for developing anti-inflammatory drugs in search of a better therapeutic and quality of life for the patient [20].

In the course of our continuing search for bioactive natural products from plants, we have published reviews of extracts and compounds derived from plants with the following potential activities: inhibitors of mammary, uterine cervical and ovarian neoplasia [21–23]; inhibitors of HMG CoA reductase, angiotensina-converting enzyme and the enzyme acetylcholinesterase [24–26]; with central analgesic activity [27]; employed in prevention of osteoporosis [28]; for the treatment of Parkinson’s disease [29]; anticonvulsant and anxiety disorders [30,31]; with antileishmanial [32]; giardicidal [33]; antileprotic [34]; hypoglycemic [35] and antiinflammatory activities [36,37]; for the treatment of malaria [38]; with antiulcer activity [39–41] and effects of plant extracts on HIV-1 Protease [42]. Our group has also reviewed the medicinal and poisonous plants of the Northeastern region of Brazil [43,44], among other review articles [45–54]. So in this work, we reviewed the literature related to anti-inflammatory activity of the plants from South American countries.

## 2. Results and Discussion

It was possible in this review to list 175 species of medicinal plants with anti-inflammatory activity. Those species are distributed in 63 families of which the following stood out: Asteraceae, Fabaceae, Euphorbiaceae, Apocynaceae and Celastraceae with 37, 17, 11, 6 and 6 species, respectively, studied so far (Table 1).

The effectiveness of the plant extracts was dependent on the type of extract used, the model of inflammation induction and the organism tested. Thus, it was possible to classify the extracts as strongly active, active, weakly active, inactive and equivocal.

Different species of *Proustia* genus have been frequently used as antiinflammatory and analgesic to treat gout and rheumatic illnesses, however, there is little information about their efficacy and acute toxicity [55]. This genus accumulates sesquiterpene α-isocedrene derivatives that are typical for the subtribus Nassauviinae of the family Asteraceae [56], and a guaianolide β-d-glucopyranoside has been previously isolated from *Proustia ilicifolia* [57].

According to Delporte *et al.* (2005) [55] in the assays carried out per os crude methanol extract (GME), hexane extract (HE) and methanol extract (ME) exhibited the strongest analgesic activities similar to the reference drug (SN). In relation to the results obtained in per os anti-inflammatory studies, ME showed the strongest effect, and was similar to the reference drug (SN); HE did not present significant antiinflammatory activity. The antiinflammatory activity have been attributed the presence of compounds with a similar mechanism for both activities, as for example inhibition of the synthesis of prostaglandin E_2_ (PGE_2_). By the activation of the cyclo-oxygenase enzyme, the level of PGE_2_ increases markedly, and its production provokes inflammation and pain [58]. Therefore, we assume that some active metabolites of these extracts could inhibit cyclooxygenase activity.

For arachidonic acid (AA) and phorbol 12-myristate 13-acetate (TPA) induced oedema, GME showed significant effect only against AA assay and on the contrary, HE and ME presented important activities only against TPA and dichloromethane extract (DCE) was active in both AA and TPA models. The action’s mechanism of the GME can be explained by inhibition of cyclooxygenase enzymes while the HE and ME may act by inhibiting the synthesis of leukotrienes. Since the DCE in addition to inhibiting the synthesis of leukotrienes may act by blocking production of PGE_2_ [59].

GME did not show acute toxicity per os up to the maxim dose of 2 g/kg and the weight of the mice had a normal variation after the seven days of observation. Common side effects such as, mild diarrhea, loss of weight and depression were not recorded. It is important to carry out toxicological studies in other animal species in order to demonstrate its lack of toxicity [59].

*Ageratum conyzoides* (Asteraceae), known commonly as “mentrasto”, has been used in Brazillian folk medicine to treat various ailments (metrorrhagia, fevers, dermatitis, inflammation, rheumatism, diarrhea and diuretics). A large number of pharmacological activities (anti-inflammatory, antipyretic, analgesic) have been attributed to the essential oil of *Ageratum conyzoides* [60]. The flowers and leaves are used in the form of an infusion for their analgesic and antiinflammatory properties. Literature data indicate its efficacy in alleviating pain caused by human arthritis [61] or induced experimentally [62].

The hydroalcoholic extract (HAE) of the leaves from *A. conyzoides* was active in both the on subacute (cotton pellet-induced granuloma) and chronic (formaldehyde-induced arthritis) models of inflammation in rats. The weights of cotton pellets were significantly reduced in (38%) after treatment with crude extract of *A. conyzoides* (250 mg/kg, p. o.) and possibly this effect is related to inhibition of neutrophil migration. Exame macroscopic gastric mucosa did not reveal any tissue damage associated with treatment, which is a collateral effect of many antiinflammatory drugs, including aspirin and related compounds [63,64], this result would be explained by an inhibition of the biosynthesis of prostanoids by cyclooxygenase [65].

Literature review reports indicate the presence of pyrrolizidine alkaloids in *A. conyzoides* plants [66,67]. These are known to be hepatotoxic, and to cause lung cancer and variety of other ailments [68]. There was investigated possible hematological and biochemical alteration in animal blood samples following after sub-acute and chronic treatment with the HAE of the plant. To evaluate liver function, serum glutamic oxaloacetic transaminase (SGOT) and serum glutamic pyruvic transaminase (SGPT) levels of plasma were measured. It was observed that during the sub-acute treatment, no significant alteration in serum levels of SGOT and SGPT, however during the chronic treatment with HAE (500 mg/kg body wt.) the value of SGPT (108.5726.6 U/l) showed a statistically significant difference (*p* < 0,05) to control group (155.6739.6 U/l), reduced significantly [65].

*Artemisia* copa Phil. (Compositae), commonly known as “copa-copa”, is a small and much branched bush with a height of 30–60 cm that grows in the northwest of Argentina and in the north of Chile. The plant is regularly sold in local markets and herb health stores and the infusion of the aerial parts are used in popular medicine as antitussive, digestive, for lowering fever, for pulmonary diseases, and hypertension [69]. The leaves, macerated in alcohol, are also used locally to rub on rheumatic pains [70].

Anti-inflammatory activity of ethanol and dichloromethane extracts were analyzed in models of carrageenan-induced paw edema in rats and the ear edema induced by 12-*O*-tetradecanoylphorbol-13 acetate (TPA) and arachidonic acid (AA) in mice. Antiinflammatory activity was observed in both extracts that showed antiinflammatory activity in the TPA (88 and 54%), and the ethanolic extract showed a 37% inhibition in AA test. The results suggested that *A. copa* was able to prevent the production of proinflammatory mediators specially those related with cyclooxygenase (CO) and Lipoxygenase (LO) pathway. *A. copa* has no analgesic effect on the central nervous system that would contribute to its peripheral analgesic effect [71].

*Bauhinia tarapotensis* Benth. (Leguminosae) is a small tree growing in Ecuador (South America), where it is commonly known as “pata de vaca”. The plant leaves are traditionally used for their anti-inflammatory and decongestant properties [72], whereas the bark is employed as antidiarrhoeal remedy [73]. Previous study on the methanol extract of *B. tarapotensis* leaves revealed antioxidant and radical scavenger properties, due to the presence of different antioxidant principles, such as cyclohexenone, lignans, and phenylethanoids derivatives [74].

The topical anti-inflammatory activity was evaluated as inhibition of the croton oil-induced ear edema in mice [75]. Five extracts of the leaves significantly inhibited the croton oil-induced ear edema in mice, among which the chloroform extract was the most active. The main anti-inflammatory principles of *B. tarapotensis* leaves are triterpenic acids of ursane and oleanane series. The antiphlogistic activity of mixtures constituted of two ursane and oleanane isomers with different hydroxylation pattern, in the ratio 2:1, is comparable to that of indomethacin [76].

*Croton pullei* (Euphorbiaceae) is a liana that grows above other trees, distributed in tropical areas with vast distribution in the Amazon forest [77]. In the folk medicine, several plants of the *Croton* genus have been used with therapeutic purposes in pathologies that involve painful and inflammatory diseases which justify this work [78].

Anti-inflammatory activity was tested in two models that assess inflammatory processes such as edema and leukocyte migration. The crude methanol extract significantly reduced by 72% the ear edema by croton-oil induced, as also was a dose-dependent reduction of leukocyte migration to the peritoneum after induction with carrageenan. The mechanism of action has not yet elucidated [78].

*Maytenus ilicifolia* Mart. ex. Reiss (Celastraceae), popularly called “espinheira-santa” due to the appearance of its leaves and its therapeutic properties, is utilized in popular medicine in cases of inflammation and gastric ulcer [79–81].

This study evaluated the anti-inflammatory activity, antinociceptive and antiulcer of ethyl acetate and hexane extracts of *Maytenus ilicifolia* [82].

In the model of paw edema induced by carrageenan was observed that there was no significant difference in inflammatory response between indomethacin and the extracts evaluated. The result of hexane extract showed the anti-inflammatory potential of terpenes whereas for ethylacetate extract the anti-inflammatory response has been attributed to flavonoids, which act by reducing the formation of pro-inflammatory mediators as prostaglandins, leukotrienes, reactive oxygen species and nitric oxide [82]. According to Oliveira *et al*. (1991) [83], both acute and chronic administration of this species did not induce any apparent toxicity.

## 3. Material and Methods

In the present work, the anti-inflammatory activity of the plants was searched through the data bank of the University of Illinois in Chicago, the NAPRALERT (Acronym for Natural Products ALERT). The data were updated in September 2009, using anti-inflammatory plants as legend. The plant extracts studied in South America were selected for this work and the references found in the search were later consulted for details of the models or mechanisms.

## 4. Conclusion

Given the above, this review is of fundamental importance to intensify studies with medicinal plants for the discovery of new bioactive molecules in healing of many diseases, including inflammation, thus benefiting populations affected by ensuring a better quality of life.

## Figures and Tables

**Table 1.** Extracts of plants with anti-inflammatory activity studied in South America.

**Family and Botanical name**	**Country**	**Part used**	**Type of extract**	**Model assay/way of route**	**Organism tested**	**Activity**	**Ref.**
Acanthaceae							
*Justicia pectoralis var. stenophylla*	Brazil	Dried leaf	Hydro-alcoholic ext	Carrageenan-induced pedal edema/Intragastric	Rat	Active	[84]
	Brazil	Dried leaf	Hexane-acetone	Dextran-induced pedal edema/Intragastric	Rat	Inactive	[84]
Agavaceae							
*Cordyline dracaenoides*	Brazil	Dried rhizome	EtOH-H_2_O (50%) ext	Carrageenan-induced pedal edema/IP	Rat	Active	[85]
Alismataceae							
*Echinodorus grandiflorus*	Brazil	Dried rhizome	MeOH ext	Carrageenan-induced pedal edema/Intragastric	Mouse	Active	[86]
	Brazil	Dried rhizome	MeOH ext	Carrageenan-induced pedal edema/Intragastric	Rat	Active	[86]
Amaranthaceae							
*Alternanthera brasiliana*	Brazil	Dried leaf	H_2_O ext	Carrageenan-induced pedal edema/Route not given	Rat	Inactive	[87]
*Pfaffia glomerata*	Brazil	Dried root	EtOH (60%) ext	Acetic acid-induced pedal edema/Intragastric	Mouse	Active	[88]
	Brazil	Dried root	EtOH (60%) ext	Acetic acid-induced pedal edema/IP	Mouse	Active	[88]
*Pfaffia iresinoides*	Brazil	Dried root	Saponin fraction	Carrageenan-induced pleurisy/Intragastric	Rat	Active	[89]
	Brazil	Dried root	H_2_O ext	Carrageenan-induced pleurisy/Intragastric	Rat	Active	[89]
	Brazil	Dried root	H_2_O ext	Cotton pellet granuloma/Intragastric	Rat	Inactive	[89]
	Brazil	Dried root	Saponin fraction	Cotton pellet granuloma/Intragastric	Rat	Active	[89]
*Pfaffia paniculata*	Brazil	Dried root	EtOH (20%) ext	Carrageenan-induced pedal edema/IP	Mouse	Active	[90]
	Brazil	Dried root	EtOH (60%) ext	Carrageenan-induced pedal edema/IP	Rat	Active	[91]
*Pfaffia stenophylla*	Brazil	Dried root	EtOH (20%) ext	Carrageenan-induced pedal edema/IP	Mouse	Active	[90]
Anacardiaceae							
*Anacardium occidentale*	Brazil	Dried bark	Shell	Carrageenan-induced pedal edema/Gastric intubation	Rat	Active	[92]
	Brazil	Dried bark	Shell	Dextran-induced pedal edema/Gastric intubation	Rat	Inactive	[92]
	Brazil	Dried bark	Shell	Cotton pellet granuloma/IP	Rat	Active	[92]
	Brazil	Dried bark	Shell	Dextran-induced pedal edema/IP	Rat	Active	[92]
	Brazil	Dried bark	Shell	Carrageenan-induced pedal edema/IP	Rat	Active	[92]
	Brazil	Dried bark	Isopropanol-H_2_O (1:1) ext	Carrageenan-induced pedal edema/IP	Rat	Active	[92]
	Brazil	Dried bark	Shell	Number of leukocytes in exudate/IP	Rat	Active	[92]
*Astronium urundeuva*	Brazil	Stembark	EtoAc ext	Carrageenan-induced pedal edema/Intragastric	Mouse	Active	[93]
	Brazil	Dried bark	Tannin fraction	Dextran-induced pedal edema/IP	Rat	Active	[93]
	Brazil	Dried bark	Tannin fraction	Carrageenan-induced pedal edema/IP	Mouse	Active	[93]
	Brazil	Dried bark	Tannin fraction	Cyclophosphamide-induced hemorrhagic cystitis/IP	Rat	Active	[93]
	Brazil	Stembark	EtoAc ext	Carrageenan-induced pedal edema/IP	Mouse	Active	[93]
*Spondias mombin*	Venezuela	Dried bark	EtOH (100%) ext	Carrageenan-induced pedal edema/Intragastric	Rat	Active	[94]
Apocynaceae							
*Bonafousia longituba*	Ecuador	Dried part not specified	EtOH (100%) ext	Carrageenan-induced pedal edema/Intragastric	Mouse	Active	[95]
	Ecuador	Dried entire plant	EtOH (100%) ext	Carrageenan-induced pedal edema/Intragastric	Mouse	Weak activity	[95]
	Ecuador	Dried part not specified	CH_2_Cl_2_ ext	Carrageenan-induced pedal edema/Intragastric	Mouse	Active	[96]
	Ecuador	Dried part not specified	CH_2_Cl_2_ ext	Carrageenan-induced pedal edema/Intragastric	Mouse	Active	[96]
*Ervatamia coronaria*	Brazil	Dried stem	EtOH (95%) ext	Carrageenan-induced pedal edema/IP	Rat	Active	[97]
	Brazil	Dried stem	EtOH (95%) ext	Carrageenan-induced pedal edema/Intragastric	Rat	Active	[97]
	Brazil	Dried stem	H_2_O ext	Carrageenan-induced pedal edema/IP	Rat	Active	[97]
*Himatanthus sucuuba*	Brazil	Latex (unspec part)	Hexane ext	Carrageenan-induced pedal edema/Intragastric	Rat	Active	[98]
*Mandevilla velutina*	Brazil	Dried rhizome	Aqueous-alcoholic ext	Carrageenan-induced pedal edema/Intragastric	Mouse	Active	[99]
	Brazil	Dried rhizome	Aqueous-alcoholic ext	Carrageenan-induced pedal edema/Intragastric	Rat	Active	[99]
	Brazil	Dried rhizome	Aqueous-alcoholic ext	5-HT-induced pedal edema/Intragastric	Rat	Inactive	[99]
	Brazil	Dried rhizome	Aqueous-alcoholic ext	Carrageenan-induced pedal edema/IP	Rat	Active	[99]
	Brazil	Dried rhizome	Aqueous-alcoholic ext	Snake venom-induced pedal edema/Intragastric	Rat	Inactive	[99]
	Brazil	Dried rhizome	Aqueous-alcoholic ext	Platelet aggregating factor-acether induced pedal edema/Intragastric	Rat	Inactive	[99]
	Brazil	Dried entire plant	EtOH (95%) ext	Arachidonic-acid induced ear edema/Intragastric	Mouse	Active	[100]
	Brazil	Frozen rhizome	EtOH H_2_O (50%) ext	Bradykinin-induced pedal edema/Intragastric	Rat	Active	[101]
	Brazil	Frozen rhizome	EtOH H_2_O (50%) ext	Carrageenan-induced pedal edema/5-HT-induced pedal edema/Intragastric	Rat	Active	[101]
	Brazil	Frozen rhizome	EtOH H_2_O (50%) ext	Dextran-induced pedal edema/Intragastric	Rat	Active	[101]
	Brazil	Frozen rhizome	EtOH H_2_O (50%) ext	Carrageenan-induced pedal edema/IP	Rat	Active	[101]
	Brazil	Frozen rhizome	EtOH H_2_O (50%) ext	Cellulose sulfate induced rat paw edema/Intragastric	Rat	Active	[101]
	Brazil	Frozen rhizome	EtOH H_2_O (50%) ext	Paltelet aggretating factor-acether induced rat paw edema/Intragastric	Rat	Active	[101]
	Brazil	Frozen rhizome	EtOH H_2_O (50%) ext	Zymosan indued rat paw edema/Intragastric	Rat	Active	[101]
	Brazil	Frozen rhizome	EtOH H_2_O (50%) ext	Bothrops jararaca induced rat paw edema/Intragastric	Rat	Inactive	[101]
*Peschiera australis* var. *australis*	Brazil	Dried leaf	EtOH (100%) ext	Carrageenan-induced paw edema/IP	Rat	Active	[102]
	Brazil	Dried leaf	H_2_O ext	Carrageenan-induced paw edema/IP	Rat	Active	[102]
*Peschiera vanheurckii*	Peru	Dried stembark	EtOH (100%) ext	EPP-induced rat ear oedema/External	Rat	Active	[103]
Araliaceae							
*Hedera helix*	Uruguay	Dried leaf	EtOH (95%) ext	**/Oral	Human adult	Active	[104]
Arecaceae							
*Orbignya phalerata*	Brazil	Dried fruit	CHCl_3_ ext	Cotton pellet granuloma/Intragastric	Rat	Active	[105]
	Brazil	Dried fruit	CHCl_3_ ext	Carrageenan-induced pedal edema/Intragastric	Rat	Active	[105]
	Brazil	Dried fruit	CHCl_3_ ext	Carrageenan-induced pedal edema/IP	Rat	Active	[105]
Aristolochiaceae							
*Aristolochia triangularis*	Argentina	Dried root	MeOH ext	Croton oil-induced edema/External	Mouse	Active	[106]
	Argentina	Dried root	CH_2_Cl_2_ ext	Croton oil-induced edema/External	Mouse	Active	[106]
	Argentina	Dried root	H_2_O ext	Croton oil-induced edema/External	Mouse	Active	[106]
	Argentina	Dried root	H_2_O ext	Carrageenan-induced pedal edema/IP	Mouse	Active	[106]
	Argentina	Dried root	CH_2_Cl_2_ ext	Carrageenan-induced pedal edema/IP	Mouse	Active	[106]
	Argentina	Dried root	MeOH ext	Carrageenan-induced pedal edema/IP	Mouse	Inactive	[106]
Asclepiadaceae							
*Marsdenia cundurango*	Ecuador	Part not specified	CH_2_Cl_2_ ext	Carrageenan-induced pedal edema/Intragastric	Mouse	Active	[96]
	Ecuador	Dried entire plant	EtOH (100%) ext	Carrageenan-induced pedal edema/Intragastric	Mouse	Active	[95]
Asteraceae							
*Achyrocline satureioides*	Brazil	Dried inflorescence	H_2_O ext	Carrageenan-induced pedal edema/IP	Rat	Active	[107]
	Brazil	Dried inflorescence	EtOH (95%) ext	Carrageenan-induced pedal edema/IP	Rat	Active	[107]
	Brazil	Dried inflorescence	Hot H_2_O ext	Carrageenan-induced pedal edema/IP	Rat	Active	[107]
	Brazil	Dried inflorescence	EtOH (95%) ext	Croton oil ear edema test/External	Mouse	Active	[107]
	Brazil	Dried inflorescence	H_2_O ext	Croton oil ear edema test/External	Mouse	Active	[107]
	Brazil	Dried inflorescence	Hot H_2_O ext	Croton oil ear edema test/External	Mouse	Active	[107]
*Ageratum conyzoides*	Brazil	Dried leaf	EtOH (70%) ext	Formalin-induced pedal edema/Intragastric	Rat	Active	[62]
	Brazil	Dried leaf	EtOH (70%) ext	Cotton pellet granuloma/Intragastric	Rat	Active	[62]
	Brazil	Dried leaf	EtOH (70%) ext	Carrageenan-induced pedal edema/SC	Rat	Active	[62]
	Brazil	Dried leaf	Hydro-alcoholic ext	Formalin-induced pedal edema/Intragastric	Rat	Active	[65]
	Brazil	Dried leaf	Hydro-alcoholic ext	Cotton pellet granuloma/Intragastric	Rat	Active	[65]
	Brazil	Dried leaf	EtOH (70%) ext	Yeast-induced inflammation of the paw/IP	Rat	Active	[108]
*Ambrosia tenuifolia*	Argentina	Dried aerial parts	CH_2_Cl_2_ ext	Croton oil-induced edema/External	Mouse	Active	[106]
	Argentina	Dried aerial parts	MeOH ext	Croton oil-induced edema/External	Mouse	Active	[106]
	Argentina	Dried aerial parts	H_2_O ext	Croton oil-induced edema/External	Mouse	Active	[106]
	Argentina	Dried aerial parts	MeOH ext	Carrageenan-induced pedal edema/IP	Mouse	Inactive	[106]
	Argentina	Dried aerial parts	H_2_O ext	Carrageenan-induced pedal edema/IP	Mouse	Active	[106]
	Argentina	Dried aerial parts	CH_2_Cl_2_ ext	Carrageenan-induced pedal edema/IP	Mouse	Active	[106]
*Artemisia copa*	Argentina	Dried entire plant	Hot H_2_O ext	Carrageenan-induced pedal edema/IP	Rat	Inactive	[109]
	Argentina	Dried entire plant	CH_2_Cl_2_ ext	Carrageenan-induced pedal edema/IP	Rat	Inactive	[109]
	Argentina	Dried entire plant	MeOH ext	Carrageenan-induced pedal edema/IP	Rat	Inactive	[109]
	Argentina	Dried aerial parts	H_2_O ext	12-*O*-tetradecanoylphorbol-13-acetate (TPA)-induced ear inflammation/External	Mouse	Active	[71]
	Argentina	Dried aerial parts	CH_2_Cl_2_ ext	12-*O*-tetradecanoylphorbol-13-acetate (TPA)-induced ear inflammation/External	Mouse	Active	[71]
*Baccharis articulata*	Argentina	Dried aerial parts	H_2_O ext	Carrageenan-induced pedal edema/Intragastric	Rat	Inactive	[110]
	Argentina	Dried aerial parts	H_2_O ext	Carrageenan-induced pedal edema/IP	Rat	Inactive	[110]
*Baccharis crispa*	Argentina	Dried aerial parts	H_2_O ext	Carrageenan-induced pedal edema/Intragastric	Rat	Inactive	[110]
	Argentina	Dried aerial parts	H_2_O ext	Carrageenan-induced pedal edema/IP	Rat	Active	[110]
*Baccharis decussata*	Colombia	Dried leaf	MeOH ext	**/Route not given	**	Active	[111]
*Baccharis incarum*	Argentina	Dried entire plant	CH_2_Cl_2_ ext	Carrageenan-induced pedal edema/IP	Rat	Active	[109]
	Argentina	Dried entire plant	MeOH ext	Carrageenan-induced pedal edema/IP	Rat	Inactive	[109]
	Argentina	Dried entire plant	Hot H_2_O ext	Carrageenan-induced pedal edema/IP	Rat	Inactive	[109]
*Baccharis medullosa*	Argentina	Dried aerial parts	CHCl_3_ ext	Carrageenan-induced pedal edema/IP	Mouse	Inactive	[112]
	Argentina	Dried aerial parts	EtoAc ext	Carrageenan-induced pedal edema/IP	Mouse	Inactive	[112]
	Argentina	Dried aerial parts	CCl_4_	Carrageenan-induced pedal edema/IP	Mouse	Inactive	[112]
	Argentina	Dried aerial parts	Hexane ext	Carrageenan-induced pedal edema/IP	Mouse	Active	[112]
*Baccharis rufescens*	Argentina	Dried aerial parts	Hexane ext	Carrageenan-induced pedal edema/IP	Mouse	Inactive	[112]
	Argentina	Dried aerial parts	Acetone ext	Carrageenan-induced pedal edema/IP	Mouse	Active	[112]
	Argentina	Dried aerial parts	CHCl_3_ ext	Carrageenan-induced pedal edema/IP	Mouse	Active	[112]
*Baccharis trimera*	Uruguay	Dried aerial parts	H_2_O ext	Carrageenan-induced pedal edema/Intragastric	Rat	Inactive	[110]
	Uruguay	Dried aerial parts	H_2_O ext	Carrageenan-induced pedal edema/IP	Rat	Active	[110]
	Uruguay	Dried aerial parts	Butanol ext	Carrageenan-induced edema in rat paw/IP	Rat	Active	[113]
	Uruguay	Dried aerial parts	Butanol ext	Dextran-induced edema in rat paw/IP	Rat	Active	[113]
	Uruguay	Dried aerial parts	Butanol ext	Arachindonic acid-induced edema in pat paw/IP	Rat	Equivocal	[113]
	Uruguay	Dried aerial parts	Butanol ext	C16-PAF-induced edema/IP	Rat	Weak activity	[113]
	Uruguay	Dried aerial parts	Butanol ext	Zymosan-induced edema in rat paw/IP	Rat	Equivocal	[113]
*Baccharis trinervis*	Ecuador	Dried entire plant	EtOH (100%) ext	Carrageenan-induced pedal edema/Intragastric	Mouse	Weak activity	[95]
*Baccharis tucumanensis*	Argentina	Dried aerial parts	H_2_O ext	Croton oil-induced edema/External	Mouse	Active	[106]
	Argentina	Dried aerial parts	CH_2_Cl_2_ ext	Croton oil-induced edema/External	Mouse	Active	[106]
	Argentina	Dried aerial parts	MeOH ext	Croton oil-induced edema/External	Mouse	Active	[106]
	Argentina	Dried aerial parts	CH_2_Cl_2_ ext	Carrageenan-induced pedal edema/IP	Mouse	Inactive	[106]
	Argentina	Dried aerial parts	H_2_O ext	Carrageenan-induced pedal edema/IP	Mouse	Active	[106]
	Argentina	Dried aerial parts	MeOH ext	Carrageenan-induced pedal edema/IP	Mouse	Active	[106]
*Bidens pilosa*	Brazil	Dried leaf	MeOH ext	Zymosan-induced pedal edema/IP	Mouse	Active	[114]
*Bidens subalternans*	Argentina	Dried entire plant	MeOH ext	Carrageenan-induced pedal edema/IP	Mouse	Weak activity	[115]
	Argentina	Dried entire plant	CHcl_3_ ext	Carrageenan-induced pedal edema/IP	Mouse	Active	[115]
*Centaurea chilensis*	Chile	Dried aerial parts	MeOH ext	**/Intragastric	Guinea pig	Active	[116]
	Chile	Dried aerial parts	CHCl_3_ ext	**/Intragastric	Guinea pig	Active	[116]
*Chromolaena christieana*	Argentina	Dried aerial parts	MeOH ext	Croton oil-induced edema/External	Mouse	Active	[106]
	Argentina	Dried aerial parts	CH_2_Cl_2_ ext	Croton oil-induced edema/External	Mouse	Active	[106]
	Argentina	Dried aerial parts	H_2_O ext	Croton oil-induced edema/External	Mouse	Active	[106]
	Argentina	Dried aerial parts	CH_2_Cl_2_ ext	Carrageenan-induced pedal edema/IP	Mouse	Inactive	[106]
	Argentina	Dried aerial parts	H_2_O ext	Carrageenan-induced pedal edema/IP	Mouse	Inactive	[106]
	Argentina	Dried aerial parts	MeOH ext	Carrageenan-induced pedal edema/IP	Mouse	Inactive	[106]
*Conyza bonariensis*	Brazil	Aerial part essent oil	Essential oil	LPS-induced leukocyte recruitement/Intragastric	Mouse	Active	[117]
*Conyza floribunda*	Ecuador	Dried entire plant	EtOH (100%) ext	Carrageenan-induced pedal edema/Intragastric	Mouse	Weak activity	[95]
*Conyza sophiifolia*	Argentina	Dried aerial parts	Hexane ext	Paw edema test/Route not given	Rat	Active	[118]
	Argentina	Dried aerial parts	Acetone ext	Paw edema test/Route not given	Rat	Active	[118]
	Argentina	Dried aerial parts	CHCl_3_ ext	Paw edema test/Route not given	Rat	Active	[118]
*Cynara scolymus*	Brazil	Fresh leaf	Infusion	Dye diffusion assay/Intragastric	Mouse	Active	[119]
*Elephantopus scaber*	Brazil	Fresh leaf	Infusion	Dye diffusion assay/Intragastric	Mouse	Active	[119]
	Brazil	Dried entire plant	EtOH-H_2_O (50%) ext	Carrageenan-induced pedal edema/Intragastric	Rat	Inactive	[120]
	Brazil	Dried entire	Decoction	Carrageenan-induced pedal edema/Intragastric	Rat	Inactive	[120]
*Eremanthus erythropappus*	Brazil	Dried aerial parts	EtOH (95%) ext	Carrageenan-induced pedal edema/Intragastric	Rat	Active	[121]
*Eupatorium buniifolium*	Argentina	Dried aerial parts	CH_2_Cl_2_ ext	12-*O*-tetradecanoylphorbol-13-acetate (TPA)-induced ear inflammation/External	Mouse	Active	[122]
	Equador	Dried entire plant	EtOH (100%) ext	Carrageenan-induced pedal edema/Intragastric	Mouse	Active	[95]
*Eupatorium inulaefolium* var*. suaveolens*	Argentina	Oven dried aerial parts	CH_2_Cl_2_ ext	Phorbol myristate acetate-induced ear	Mouse	Active	[123]
				Inflammation/External			
	Argentina	Oven dried aerial parts	CH_2_Cl_2_ ext	Carrageenan-induced pedal edema/Intragastric	Rat	Active	[123]
*Gochnatia polymorpha*	Brazil	Dried leaf	H_2_O ext	Carrageenan-induced pedal edema/Intragastric	Rat	Active	[124]
	Brazil	Dried leaf	EtOH (100%) ext	Carrageenan-induced pedal edema/Intragastric	Rat	Active	[124]
	Brazil	Dried leaf	Butanol ext	Carrageenan-induced pedal edema/Intragastric	Rat	Inactive	[124]
	Brazil	Dried leaf	EtoAc ext	Carrageenan-induced pedal edema/Intragastric	Rat	Active	[124]
	Brazil	Dried leaf	Dichloromethane ext	Carrageenan-induced pedal edema/Intragastric	Rat	Inactive	[124]
*Laennecia sophiifolia*	Argentina	Dried aerial parts	Hexane ext	Carrageenan-induced pedal edema/IP	Mouse	Active	[112]
	Argentina	Dried aerial parts	Acetone ext	Carrageenan-induced pedal edema/IP	Mouse	Active	[112]
*Mikania glomerata*	Brazil	Fresh leaf	Infusion	Dye diffusion assay/Intragastric	Mouse	Active	[119]
	Brazil	Dried leaf	EtOH-H_2_O (1:1) ext	PAF-induced edema/Histamine-induced edema/SC	Rat	Inactive	[125]
	Brazil	Dried leaf	Dichloromethanol	Carrageenan-induced pleurisy/IP	Mouse	Active	[126]
	Brazil	Dried leaf	EtOH-H_2_O (1:1) ext	Serotonin-induced pleural edema/SC	Rat	Inactive	[125]
*Mutisia kurtzii*	Argentina	Dried entire plant	CH_2_Cl_2_ ext	Carrageenan-induced pedal edema/IP	Rat	Inactive	[109]
	Argentina	Dried entire plant	MeOH ext	**/IP	Rat	Inactive	[109]
	Argentina	Dried entire plant	Hot H_2_O ext	Carrageenan-induced pedal edema/IP	Rat	Active	[109]
*Neurolaena lobata*	Ecuador	Dried entire plant	EtOH (100%) ext	Carrageenan-induced pedal edema/Intragastric	Mouse	Active	[95]
*Pluchea sagittalis*	Argentina	Dried entire plant	Hot H_2_O ext	Carrageenan-induced pedal edema/IP	Rat	Active	[123]
	Argentina	Dried entire plant	CH_2_Cl_2_ ext	Carrageenan-induced pedal edema/IP	Rat	Active	[123]
	Argentina	Oven dried aerial parts	CH_2_Cl_2_ ext	Phorbol myristate acetate-induced ear inflammation/External	Mouse	Active	[109]
	Argentina	Oven dried aerial parts	CH_2_Cl_2_ ext	Carrageenan-induced pedal edema/Intragastric	Rat	Active	[109]
	Argentina	Dried entire plant	MeOH ext	Carrageenan-induced pedal edema/IP	Rat	Inactive	[109]
*Porophyllum ruderale*	Brazil	Aerial part essent oil	Essential oil	LPS-induced leukocyte recruitement/Intragastric	Mouse	Active	[117]
*Proustia pyrifolia*	Chile	Dried aerial parts	CH_2_Cl_2_ ext	Acetic acid-induced pedal edema/External	Mouse	Active	[59]
	Chile	Dried aerial parts	CH_2_Cl_2_ ext	12-*O*-tetradecanoylphorbol-13-acetate (TPA)-induced ear inflammation/External	Mouse	Active	[59]
	Chile	Dried aerial parts	MeOH ext	12-*O*-tetradecanoylphorbol-13-acetate (TPA)-induced ear inflammation/External	Mouse	Active	[59]
	Chile	Dried aerial parts	MeOH ext	Acetic acid-induced pedal edema/External	Mouse	Active	[59]
	Chile	Dried aerial parts	Hexane ext	Acetic acid-induced pedal edema/External	Mouse	Active	[59]
	Chile	Dried aerial parts	Hexane ext	12-*O*-tetradecanoylphorbol-13-acetate (TPA)-induced ear inflammation/External	Mouse	Active	[59]
*Synedrella nodiflora*	Venezuela	Dried leaf	EtOH (100%) ext	Carrageenan-induced pedal edema/Intragastric	Rat	Active	[94]
	Venezuela	Dried leaf	Hexane ext	Carrageenan-induced pedal edema/Intragastric	Rat	Active	[94]
*Tagetes pusilla*	Ecuador	Dried entire plant	EtOH (100%) ext	Carrageenan-induced pedal edema/Intragastric	Mouse	Active	[95]
*Tanacetum vulgare*	Argentina	Dried aerial parts	Dichloromethane ext	12-*O*-tetradecanoylphorbol-13-acetate (TPA)-induced ear inflammation/External	Mouse	Active	[127]
	Argentina	Dried aerial parts	EtOH (100%) ext	12-*O*-tetradecanoylphorbol-13-acetate (TPA)-induced ear inflammation/External	Mouse	Weak activity	[128]
	Argentina	Dried aerial parts	CHCl_3_ ext	12-*O*-tetradecanoylphorbol-13-acetate (TPA)-induced ear inflammation/External	Mouse	Active	[128]
*Vanillosmopsis arborea*	Brazil	Dried trunkwood	Essential oil	**/Gastric intubation	Mouse	Active	[129]
Bignoniaceae							
*Adenocalymma alliacea*	Peru	Dried root + stem	EtOH (100%) ext	EPP-induced rat ear oedema/External	Rat	Weak activity	[103]
*Tabebuia impetiginosa*	Brazil	Dried bark	Type ext not stated	Formalin-induced pedal edema/Route not given	Rat	Active	[130]
*Tecoma sambucifolia*	Peru	Dried flowers	H_2_O ext	Carrageenan-induced pedal edema/IP	Rat	Active	[131]
	Peru	Dried flowers	EtOH (95%) ext	Carrageenan-induced pedal edema/IP	Rat	Active	[131]
	Peru	Dried perianth	H_2_O ext	Carrageenan-induced pedal edema/IP	Rat	Active	[131]
	Peru	Dried perianth	EtOH (95%) ext	Carrageenan-induced pedal edema/IP	Rat	Active	[131]
*Tynnanthus myrianthus*	Peru	Dried part not specified	EtOH (95%) ext	Carrageenan-induced pedal edema/IP	Rat	Active	[132]
Boraginaceae							
*Auxemma oncocalyx*	Brazil	Dried heartwood	Quinone fraction	Carrageenan-induced pedal edema/Intragastric	Rat	Active	[133]
	Brazil	Dried heartwood	Quinone fraction	Dextran-induced pedal edema/IP	Rat	Active	[133]
	Brazil	Dried heartwood	Quinone fraction	Carrageenan-induced pedal edema/IP	Rat	Active	[133]
*Cordia verbenacea*	Brazil	Freeze-dried leaf	Lyophilized extract	Miconazole-induced edema/Intragastric	Rat	Active	[134]
	Brazil	Freeze-dried leaf	Lyophilized extract	Nystatin-induced edema/External	Rat	Active	[134]
	Brazil	Fresh leaf	EtOH (70%) ext	Cotton pellet granuloma/External	Rat	Active	[135]
	Brazil	Fresh leaf	EtOH (70%) ext	Cotton pellet granuloma/Intragastric	Rat	Active	[135]
	Brazil	Dried leaf	EtOH (70%) ext	Croton oil-induced edema/External	Mouse	Active	[136]
	Brazil	Dried leaf	EtOH (70%) ext	Nystatin-induced pedal edema/Gastric intubation	Rat	Active	[136]
	Brazil	Dried leaf	EtOH (70%) ext	Cold stress and carrageenin-induced edema combined/Gastric intubation	Rat	Active	[136]
	Brazil	Fresh leaf	EtOH (70%) ext	Carrageenan-induced pedal edema/Oral	Rat	Active	[137]
	Brazil	Fresh leaf	EtOH (70%) ext	Cotton pellet granuloma/Oral	Rat	Active	[137]
	Brazil	Freeze-dried leaf	Lyophilized extract	Nystatin-induced edema/External	Rat	Active	[134]
*Symphytum officinale*	Brazil	Dried leaf	Aqueous high speed supernatant	Carrageenan-induced pedal edema/Gastric intubation	Rat	Inactive	[138]
Bromeliaceae							
*Nidularium procerum*	Brazil	Dried leaf	H_2_O ext	LPS-induced inflammatory/IP	Mouse	Active	[139]
*Tillandsia streptocarpa*	Brazil	Dried entire plant	MeOH ext	Croton oil-induced edema/External	Mouse	Active	[140]
Burseraceae							
*Bursera simaruba*	Venezuela	Dried leaf	Hexane ext	Carrageenan-induced pedal edema/Intragastric	Rat	Active	[94]
	Venezuela	Dried leaf	EtOH (100%) ext	Carrageenan-induced pedal edema/Intragastric	Rat	Active	[94]
	Venezuela	Dried bark	EtOH (100%) ext	Carrageenan-induced pedal edema/Intragastric	Rat	Active	[94]
	Venezuela	Dried leaf	Hexane ext	Carrageenan-induced pedal edema/Intragastric	Rat	Active	[141]
*Protium kleinii*	Brazil	Dried bark	Ether ext	Arachidonic acid-induced edema/External	Mouse	Inactive	[142]
	Brazil	Dried bark	Ether ext	12-*O*-tetradecanoylphorbol-13-acetate (TPA)-induced ear inflammation/External	Mouse	Active	[142]
Celastraceae							
*Cheiloclinium cognatum*	Brazil	Dried rootbark	MeCl_2_ ext	Croton oil-induced edema/Intragastric	Mouse	Active	[143]
*Maytenus aquifolium*	Brazil	Dried leaf/plus piroxican	Hydro-alcoholic ext	Carrageenan-induced pedal edema/Intragastric	Rat	Active	[144]
	Brazil	Dried leaf	Hydro-alcoholic ext	Carrageenan-induced pedal edema/Intragastric	Rat	Active	[144]
*Maytenus boaria*	Chile	Dried aerial parts	MeOH ext	Carrageenan-induced pedal edema/Intragastric	Guinea pig	Active	[145]
*Maytenus ilicifolia*	Brazil	Dried leaf	Hexane-acetone	**/Intragastric	Rat	Active	[82]
	Brazil	Dried leaf	EtoAc ext	Carrageenan-induced pedal edema/Intragastric	Rat	Active	[82]
	Brazil	Dried leaf	Hexane-acetone	Formalin-induced pedal edema/Intragastric	Mouse	Active	[82]
	Brazil	Dried leaf	EtoAc ext	Formalin-induced pedal edema/Intragastric	Mouse	Active	[82]
*Maytenus laevis*	Colombia	Bark	EtOH (95%) ext	Carrageenin-induced pedal edema/SC	Rat	Active	[146]
*Maytenus rigida*	Brazil	Dried bark	EtOH (95%) ext	Carrageenan-induced pedal edema/Intragastric	Rat	Active	[147]
Chloranthaceae							
*Hedyosmum bonplandianum*	Colombia	Dried leaf	Butanol ext	Carrageenan-induced pedal edema/Intragastric	Mouse	Active	[148]
Clusiaceae							
*Hypericum brasiliense*	Brazil	Dried leaf	Type ext not stated	Carrageenan-induced pedal edema/Oral	Rat	Active	[149]
Convolvulaceae							
*Cuscuta chilensis*	Chile	Dried entire plant	MeOH ext	Carrageenan-induced pedal edema/Intragastric	Guinea pig	Active	[150]
	Chile	Dried entire plant	Infusion	Carrageenan-induced pedal edema/Intragastric	Guinea pig	Active	[150]
*Ipomoea fistulosa*	Argentina	Oven dried aerial parts	MeOH ext	Carrageenan-induced pedal edema/Intragastric	Rat	Active	[123]
	Argentina	Oven dried aerial parts	CH_2_Cl_2_ ext	Phorbol myristate acetate-induced ear inflammation/External	Mouse	Active	[123]
Crassulaceae							
*Bryophyllum calcinum*	Brazil	Dried leaf	Lyophilized extract	Carrageenan-induced pedal edema/Intragastric	Rat	Active	[151]
*Kalanchoe brasiliensis*	Brazil	Fresh leaf	Plant juice	Carrageenan-induced pedal edema/Intragastric	Rat	Active	[152]
	Brazil	Fresh leaf	Juice	Zymosan-induced inflammation/IP	Mouse	Active	[153]
	Brazil	Fresh fruit juice (unripe)	Juice	**/IP	Mouse	Active	[154]
Cucurbitaceae							
*Cayaponia tayuya*	Brazil	Dried root	MeOH ext	Carrageenan-induced pedal edema/Gastric intubation	Mouse	Inactive	[155]
	Brazil	Dried root	CHCl_3_ ext	Carrageenan-induced pedal edema/Gastric intubation	Mouse	Weak activity	[155]
	Brazil	Dried root	MeOH ext	Carrageenan-induced pedal edema/IP	Mouse	Weak activity	[155]
	Brazil	Dried root	CHCl_3_ ext	Carrageenan-induced pedal edema/IP	Mouse	Active	[155]
	Brazil	Dried root	Infusion	Dye diffusion assay/Intragastric	Mouse	Equivocal	[119]
*Wilbrandia ebracteata*	Brazil	Dried root	CH_2_Cl_2_ ext	Carrageenan-induced pedal edema/Intragastric	Rat	Weak activity	[156]
	Brazil	Dried root	CHCl_3_ soluble fraction	Carrageenan-induced pleurisy/Intragastric	Mouse	Active	[156]
	Brazil	Dried root	CH_2_Cl_2_ ext	Carrageenan-induced pedal edema/IP	Rat	Active	[156]
	Brazil	Dried root	Chromatographic fraction	Carrageenan-induced pleurisy/IP	Mouse	Active	[156]
	Brazil	Dried root	CHCl_3_ soluble fraction	Carrageenan-induced pleurisy/IP	Mouse	Active	[156]
*Wilbrandia species*	Brazil	Dried rhizome	EtOH (70%) ext	Acetic acid-induced pedal edema/Intragastric	Mouse	Active	[157]
	Brazil	Dried rhizome	EtOH (70%) ext	Carrageenan-induced pedal edema/Intragastric	Rat	Active	[157]
	Brazil	Dried rhizome	EtOH (70%) ext	Carrageenan-induced granuloma/Intragastric	Rat	Active	[157]
Cyatheaceae							
*Trichipteris procera*	Peru	Inner bark	EtOH (95%) ext	**/**	Rabbit	Active	[158]
Cyperaceae							
*Mariscus pedunculatus*	Brazil	Venom	Essential oil	LPS-induced pleurisy model/Intragastric	Mouse	Active	[159]
Dilleniaceae							
*Curatella americana*	Brazil	Dried stembark	Hydro-alcoholic ext	Carrageenan-induced pedal edema/IP	Rat	Active	[160]
	Brazil	Dried stembark	Hydro-alcoholic ext	12-*O*-tetradecanoylphorbol-13-acetate(TPA)-induced ear inflammation/IP	Mouse	Active	[160]
	Brazil	Dried stembark	Hydro-alcoholic ext	Capsaicin induced mouse ear edema/IP	Mouse	Active	[160]
Equisetaceae							
*Equisetum arvense*	Brazil	Stem	EtOH - H_2_O (1:1) ext	Carrageenan-induced pedal edema/IP	Mouse	Active	[161]
Erythroxylaceae							
*Erythroxylum argentinum*	Brazil	Dried leaf	EtOH (70%) ext	Carrageenan-induced pedal edema/Intragastric	Rat	Active	[162]
	Brazil	Dried leaf	EtOH (70%) ext	Carrageenan-induced pedal edema/IP	Rat	Active	[162]
Euphorbiaceae							
*Alchornea castaneaefolia*	Peru	Dried part not specified	EtOH (95%) ext	Carrageenan-induced pedal edema/IP	Rat	**	[132]
	Peru	Dried stembark	EtOH (100%) ext	Epp-induced rat ear edema/**	Rat	Active	[103]
*Croton cajucara*	Brazil	Bark essential oil	Essential oil	Carrageenan-induced pedal edema/Intragastric	Mouse	Active	[163]
	Brazil	Bark essential oil	Essential oil	Cotton pellet granuloma/Intragastric	Rat	Active	[163]
*Croton celtidifolius*	Brazil	Dried bark	H_2_O ext	Carrageenan-induced pedal edema/Intragastric	Mouse	Active	[164]
	Brazil	Dried bark	EtoAc ext	Carrageenan-induced pedal edema/Intragastric	Mouse	Active	[164]
	Brazil	Dried bark	EtOH (80%) ext	Carrageenan-induced pedal edema/Intragastric	Mouse	Active	[164]
	Brazil	Dried bark	Butanol ext	Carrageenan-induced pedal edema/Intragastric	Mouse	Active	[164]
	Brazil	Dried bark	EtoAc ext	Carrageenan-induced pedal edema/IP	Mouse	Active	[164]
	Brazil	Dried bark	H_2_O ext	Carrageenan-induced pedal edema/IP	Mouse	Active	[164]
	Brazil	Dried bark	Butanol ext	Carrageenan-induced pedal edema/IP	Mouse	Active	[164]
*Croton lechleri*	Peru	Fresh sap	Latex	**/Iinjection	Rat	Active	[165]
	Ecuador	Freeze-drild latex	**	Carrageenan-induced pedal edema/IP	Rat	Active	[166]
*Croton malambo*	Venezuela	Dried bark	H_2_O ext	Albumin-induced edema/IP	Mouse	Active	[167]
*Croton menthodorus*	Ecuador	Dried seed	CH_2_Cl_2_ ext	Carrageenan-induced pedal edema/Intragastric	Mouse	Active	[96]
	Ecuador	Dried entire plant	EtOH (100%) ext	Carrageenan-induced pedal edema/Intragastric	Mouse	Weak activity	[95]
*Croton pullei* var*. glabrior*	Brazil	Dried leaf	MeOH ext	Carrageenan-induced pedal edema/Intragastric	Mouse	Active	[78]
*Jatropha elliptica*	Brazil	Fresh tuber	EtOH-H_2_O(50%) ext	Carrageenan-induced pedal edema/Intragastric	Rat	Active	[168]
	Brazil	Fresh tuber	EtOH-H_2_O (50%) ext	Dextran-induced pedal edema/Intragastric	Rat	Inactive	[168]
	Brazil	Fresh tuber	EtOH-H_2_O(50%) ext	Serotonin-induced pedal edema/Intragastric	Rat	Active	[168]
*Phyllanthus amarus*	Brazil	Dried aerial parts	Hexane ext	Cfa induced edema/Intragastric	Mouse	Active	[169]
*Phyllanthus carolinensis*	Brazil	Dried entire plant	Hydro-alcoholic ext	Formalin-induced pedal edema/IP	Mouse	Active	[170]
*Phyllanthus corcovadensis*	Brazil	Dried leaf + root + stem	EtOH-H_2_O(1:1) ext	Carrageenan-induced pedal edema/vs.dextran-induced pedal edema/IP	Mouse	Inactive	[171]
Fabaceae							
*Apuleia leiocarpa*	Brazil	Fresh bark	Infusion	Dye diffusion assay/Intragastric	Mouse	Active	[119]
*Bauhinia guianensis*	Brazil	Dried stembark	CH_2_Cl_2_ ext	Dextran-induced pedal edema/IP	Rat	Active	[172]
	Brazil	Dried stembark	CH_2_Cl_2_ ext	Histamine-induced edema/IP	Rat	Inactive	[172]
	Brazil	Dried stembark	EtoAc ext	Histamine-induced edema/IP	Rat	Active	[172]
	Brazil	Dried stembark	EtoAc ext	Dextran-induced pedal edema/IP	Rat	Active	[172]
	Brazil	Dried stembark	MeOH ext	Carrageenan-induced pedal edema/IP	Rat	Active	[172]
	Brazil	Dried stembark	MeOH ext	Dextran-induced pedal edema/IP	Rat	Active	[172]
	Brazil	Dried stembark	MeOH ext	Histamine-induced edema/IP	Rat	Active	[172]
*Bauhinia tarapotensis*	Ecuador	Dried leaf	H_2_O ext	Croton oil-induced edema/**	Mouse	Active	[76]
	Ecuador	Dried leaf	Dichloromethane ext	Croton oil-induced edema/**	Mouse	Active	[76]
	Ecuador	Dried leaf	MeOH ext	Croton oil-induced edema/**	Mouse	Active	[76]
	Ecuador	Dried leaf	Hexane ext	Croton oil-induced edema/**	Mouse	Active	[76]
	Ecuador	Dried leaf	CHCl_3_ ext	Croton oil-induced edema/**	Mouse	Active	[76]
*Caesalpinia ferrea*	Brazil	Dried fruit	H_2_O ext	Carrageenan-induced pedal edema/Intragastric	Rat	Active	[173]
*Calliandra angustifolia*	Peru	Dried bark	EtOH (100%) ext	Epp-induced rat ear edema/**	Rat	Inactive	[103]
*Copaifera cearensis*	Brazil	Dried balsam	Oleoresin	Carrageenan-induced pedal edema/Intragastric	Mouse	Active	[174]
*Copaifera langsdorfii*	Brazil	Dried oleoresin	Resin	Acetic acid-induced colitis/Intragastric	Rat	Active	[174]
*Copaifera species*	Brazil	Oleoresin	Oleoresin	Carrageenan-induced pedal edema/Intragastric	Rat	Active	[174]
	Brazil	Oleoresin	Oleoresin	Cotton pellet granuloma/Intragastric	Rat	Active	[174]
	Brazil	Oleoresin	Oleoresin	Histamine-induced vascular peremability/Intragastric	Rat	Active	[174]
*Erythrina velutina*	Brazil	Dried leaf	Decoction	Carrageenan-induced pedal edema/Intragastric	Rat	Inactive	[175]
	Brazil	Dried leaf	Decoction	Carrageenan-induced pedal edema/Intragastric	Rat	Inactive	[175]
	Brazil	Dried leaf	Decoction	Carrageenan-induced pedal edema/Intragastric	Rat	Inactive	[175]
*Erythrina crista-galli*	Argentina	Dried aerial parts	Dichloromethane ext	12-*O*-tetradecanoylphorbol-13-acetate (TPA)-induced ear inflammation/**	Mouse	Active	[176]
	Argentina	Dried aerial parts	MeOH ext	12-*O*-tetradecanoylphorbol-13-acetate (TPA)-induced ear inflammation/**	Mouse	Active	[176]
	Argentina	Dried aerial parts	H_2_O ext	12-*O*-tetradecanoylphorbol-13-acetate (TPA)-induced ear inflammation/**	Mouse	Active	[176]
	Argentina	Dried aerial parts	MeOH ext	Carrageenan-induced pedal edema/Intragastric	Rat	Active	[176]
	Argentina	Dried aerial parts	H_2_O ext	Carrageenan-induced pedal edema/Intragastric	Rat	Active	[176]
	Argentina	Dried aerial parts	Dichloromethane ext	Carrageenan-induced pedal edema/Intragastric	Rat	Active	[176]
*Marsypianthes chamaedrys*	Brazil	Fresh leaf	Infusion	Dye diffusion assay/Intragastric	Mouse	Active	[119]
*Psoralea glandulosa*	Chile	Dried aerial parts	Infusion	Carrageenan-induced pedal edema/Intragastric	Guinea pig	Active	[150]
	Chile	Dried aerial parts	MeOH ext	Carrageenan-induced pedal edema/Intragastric	Guinea pig	Active	[150]
	Chile	Dried aerial parts	Pet ether ext	Carrageenan-induced pedal edema/Intragastric	Guinea pig	Active	[177]
	Chile	Dried aerial parts	Dichloromethane ext	Carrageenan-induced pedal edema/Intragastric	Guinea pig	Active	[177]
	Chile	Dried aerial parts	MeOH ext	Carrageenan-induced pedal edema/Intragastric	Guinea pig	Active	[177]
*Pterocarpus ulei*	Peru	Dried stembark	EtOH (100%) ext	EPP-induced rat ear oedema/**	Rat	Inactive	[103]
*Pterodon emarginatus*	Brazil	Dried fruit	Hexane ext	Carrageenan-induced pedal edema/Intragastric	Rat	Active	[178]
	Brazil	Dried fruit	Hexane ext	Carrageenan-induced pedal edema/Intragastric	Rat	Active	[178]
*Stryphnodendron adstringens*	Brazil	Dried stembark		Acetic acid induced vascular permeability/Intragastric	Mouse	Active	[179]
	Brazil	Dried stembark	Acetone ext	Dextran-induced pedal edema/carrageenan-induced pedal edema/Intragastric	Rat	Active	[179]
	Brazil	Dried stembark	Acetone ext	**/Intragastric	Rat	Weak activity	[179]
*Torresea cearensis*	Brazil	Dried stembark		Carrageenan-induced pedal edema/Intragastric	Rat	Active	[180]
Flacourtiaceae							
*Casearia sylvestris*	Brazil	Fresh bark + leaf	Infusion	Dye diffusion assay/Intragastric	Mouse	Weak activity	[119]
Gentianaceae							
*Gentianella achalensis*	Argentina	Dried aerial parts	Chromatographic fraction	12-*O*-tetradecanoylphorbol-13-acetate(TPA)-induced ear inflammation/**	Mouse	Active	[181]
	Argentina	Dried aerial parts	Pet ether ext	12-*O*-tetradecanoylphorbol-13-acetate(TPA)-induced ear inflammation/**	Mouse	Inactive	[181]
	Argentina	Dried aerial parts	MeOH ext	12-*O*-tetradecanoylphorbol-13-acetate(TPA)-induced ear inflammation/**	Mouse	Inactive	[181]
	Argentina	Dried aerial parts	Dichloromethane ext	12-*O*-tetradecanoylphorbol-13-acetate(TPA)-induced ear inflammation/**	Mouse	Active	[181]
	Argentina	Dried aerial parts	Dichloromethane ext	Carrageenan-induced pedal edema/Intragastric	Rat	Inactive	[181]
	Argentina	Dried aerial parts	Pet ether ext	Carrageenan-induced pedal edema/Intragastric	Rat	Inactive	[181]
	Argentina	Dried aerial parts	MeOH ext	Carrageenan-induced pedal edema/Intragastric	Rat	Inactive	[181]
Lamiaceae							
*Hyptis pectinata*	Brazil	Dried leaf	H_2_O ext	Arachidonic acid-induced edema/Intragastric	Rat	Active	[182]
	Brazil	Dried leaf	H_2_O ext	Carrageenan-induced pedal edema/Intragastric	Rat	Active	[182]
*Lavandula latifolia*	Paraguay	Aerial parts	Chromatographic fraction	Carrageenan-induced pedal edema/**	Rat	Active	[183]
	Paraguay	Aerial parts	EtOH (70%) ext	Carrageenan-induced pedal edema/**	Rat	Active	[183]
*Raphiodon echinus*	Brazil	Dried aerial parts	H_2_O ext	Acetic acid-induced dye diffusion/Intragastric	Mouse	Active	[129]
Liliaceae							
*Polygonatum punctatum*	Argentina	Oven dried aerial parts	CH_2_Cl_2_ ext	Phorbol myristate acetate-induced ear inflammation/**	Mouse	Active	[123]
	Argentina	Oven dried aerial parts	H_2_O ext	Carrageenan-induced pedal edema/Intragastric	Rat	Weak activity	[123]
Linaceae							
*Vantanea peruviana*	Peru	Dried stembark	EtOH (100%) ext	EPP-induced rat ear oedema/**	Rat	Strong activity	[103]
Loasaceae							
*Mentzelia chilensis*	Peru	Dried stem	EtoAc ext	Carrageenan-induced pedal edema/Intragastric	Rat	Active	[184]
	Peru	Dried stem	H_2_O ext	Carrageenan-induced pedal edema/Intragastric	Rat	Active	[184]
Lythraceae							
*Adenaria floribunda*	Peru	Dried stem	EtOH (100%) ext	EPP-induced rat ear oedema/**	Rat	Active	[103]
Magnoliaceae							
*Talauma ovata*	Brazil	Dried leaf	EtOH (95%) ext	Carrageenan-induced pedal edema/IP	Rat	Inactive	[185]
Malvaceae							
*Sida cordifolia*	Brazil	Dried leaf	H_2_O ext	Carrageenan-induced pedal edema/Intragastric	Rat	Active	[186]
*Urena lobata*	Ecuador	Dried entire plant	EtOH (100%) ext	Carrageenan-induced pedal edema/Intragastric	Mouse	Weak activity	[95]
Meliaceae							
*Guarea guidonia*	Brazil	Seed	EtOH (90%) ext	Carrageenan-induced pedal edema/Gastric Intubation	Rat	Active	[187]
	Brazil	Seed	EtOH (90%) ext	Cotton pellet granuloma/Gastric Intubation	Rat	Active	[187]
*Trichilia glabra*	Argentina	Dried leaf		Zymosan-induced immediate inflammation moded/IP	Mouse	Active	[188]
Menispermaceae							
*Abuta grandifolia*	Peru	Dried part not specified	EtOH (95%) ext	Carrageenan-induced pedal edema/IP	Rat	**	[132]
*Cissampelos sympodialis*	Brazil	Dried leaf	EtOH (80%) ext	Capsaicin induced edema/IP	Mouse	Active	[189]
	Brazil	Dried leaf	EtOH (80%) ext	12-*O*-tetradecanoylphorbol-13-acetate(TPA)-induced ear inflammation/IP	Mouse	Active	[189]
	Brazil	Dried leaf	EtOH (80%) ext	Carrageenan-induced pedal edema/IP	Rat	Active	[189]
Monimiaceae							
*Peumus boldus*	Chile	Dried leaf	EtOH (70%) ext	Carrageenan-induced pedal edema/IP	Rat	Active	[190]
Moraceae							
*Dorstenia brasiliensis*	Brazil	Fresh root	Infusion	Dye diffusion assay/Intragastric	Mouse	Weak activity	[119]
Myristicaceae							
*Virola pavonis*	Peru	Dried vine	EtOH (95%) ext	Carrageenan-induced pedal edema/IP	Rat	Active	[132]
*Virola peruviana*	Peru	Dried part not specified	EtOH (95%) ext	Carrageenan-induced pedal edema/IP	Rat	Active	[132]
*Eugenia uniflora*	Brazil	Fresh leaf	Infusion	Carrageenan-induced pedal edema/Intragastric	Rat	Active	[191]
	Brazil	Fresh leaf	EtOH (100%) ext	Carrageenan-induced pedal edema/Intragastric	Rat	Active	[191]
	Brazil	Fresh leaf	Decoction	Carrageenan-induced pedal edema/Intragastric	Rat	Active	[191]
	Brazil	Dried leaf	Infusion	Carrageenan-induced pedal edema/Intragastric	Rat	Inactive	[191]
	Brazil	Dried leaf	EtOH (100%) ext	Carrageenan-induced pedal edema/Intragastric	Rat	Inactive	[191]
*Psidium guineense*	Brazil	Fresh leaf essential oil	Essential oil	Carrageenan-induced pedal edema/Intragastric	Rat	Active	[192]
Olacaceae							
*Heisteria acuminata*	Ecuador	Dried part not specified	CH_2_Cl_2_ ext	Carrageenan-induced pedal edema/Intragastric	Mouse	Inactive	[96]
	Ecuador	Dried entire plant	EtOH (100%) ext	Carrageenan-induced pedal edema/Intragastric	Mouse	Active	[95]
Orchidaceae							
*Catasetum barbatum*	Paraguay	Dried aerial parts	EtOH (70%) ext	Carrageenan-induced pedal edema/**	Rat	Active	[193]
Phytolaccaceae							
*Petiveria alliacea*	Brazil	Dried root	EtOH (70%) ext	Croton oil-induced irritation/**	Rat	Active	[194]
	Brazil	Dried root	EtOH (70%) ext	Cotton pellet granuloma/**	Rat	Active	[194]
	Brazil	Dried root	Hydro-alcoholic ext	Nystatin induced edema/Intragastric	Rat	Active	[195]
	Brazil	Dried root	Hydro-alcoholic ext	Carrageenan-induced pedal edema/Intragastric	Rat	Active	[195]
	Brazil	Dried root	Lyophilized extract	Carrageenan-induced pedal edema/Intragastric	Rat	Active	[196]
	Brazil	Dried root	Hydro-alcoholic ext	Cotton pellet granuloma/Intragastric	Rat	Active	[195]
	Peru	Dried entire plant	EtOH (100%) ext	EPP-induced rat ear oedema/**	Rat	Inactive	[103]
*Phytolacca bogotensis*	Ecuador	Dried entire plant	EtOH (100%) ext	Carrageenan-induced pedal edema/Intragastric	Mouse	Inactive	[95]
	Ecuador	Dried entire plant	CH_2_Cl_2_ ext	Carrageenan-induced pedal edema/Intragastric	Mouse	Inactive	[96]
*Phytolacca rivinoides*	Ecuador	Dried entire plant	EtOH (100%) ext	Carrageenan-induced pedal edema/Intragastric	Mouse	Weak activity	[95]
	Ecuador	Dried entire plant	CH_2_Cl_2_ ext	Carrageenan-induced pedal edema/Intragastric	Mouse	Inactive	[96]
Piperaceae							
*Peperomia pellucida*	Brazil	Dried aerial	H_2_O ext	Carrageenan-induced pedal edema/Intragastric	Rat	Active	[197]
*Piper lenticellosum*	Ecuador	Dried entire plant	EtOH (100%) ext	Carrageenan-induced pedal edema/Intragastric	Mouse	Active	[95]
	Ecuador	Dried fruit	CH_2_Cl_2_ ext		Mouse	Active	[96]
*Piper marginatum*	Brazil	Dried leaf	H_2_O ext	Carrageenan-induced pedal edema/Intragastric	Rat	Active	[198]
Plantaginaceae							
*Plantago australis*	Brazil	Dried root	Hydro-alcoholic ext	Carrageenan-induced pedal edema/Intragastric	Rat	Active	[199]
	Brazil	Dried leaf	Hydro-alcoholic ext	Carrageenan-induced pedal edema/Intragastric	Rat	Active	[199]
	Brazil	Dried fruit	Hydro-alcoholic ext	Carrageenan-induced pedal edema/Intragastric	Rat	Active	[199]
*Plantago major*	Brazil	Dried leaf	H_2_O ext	Croton oil-induced edema/**	Mouse	Inactive	[200]
	Brazil	Dried leaf	H_2_O ext	Croton oil granuloma/** pouch/Intragastric	Rat	Active	[200]
	Brazil	Dried leaf	H_2_O ext	Dextran-induced pedal edema/Intragastric	Rat	Inactive	[200]
	Brazil	Dried leaf	H_2_O ext	Carrageenan-induced pleurisy/Intragastric	Rat	Active	[200]
	Brazil	Dried leaf	H_2_O ext	Carrageenan-induced pedal edema/Intragastric	Mouse	Weak activity	[200]
Polygonaceae							
*Polygonum punctatum*	Brazil	Dried entire plant	Decoction	Carrageenan-induced pedal edema/Gastric intubation	Rat	Active	[201]
	Brazil	Dried entire plant	EtOH-H_2_O (1:1) ext	Carrageenan-induced pedal edema/Gastric intubation	Rat	Active	[201]
	Brazil	Dried entire plant	EtOH-H_2_O (1:1) ext	Carrageenan-induced pedal edema/**	Rat	Inactive	[201]
	Brazil	Dried entire plant	Decoction	Carrageenan-induced pedal edema/**	Rat	Inactive	[201]
Polypodiaceae							
*Campyloneurum phyllitidis*	Paraguay	Dried leaf	H_2_O ext	Croton oil-induced edema/**	Mouse	Active	[106]
	Paraguay	Dried leaf	CH_2_Cl_2_ ext	Croton oil-induced edema/**	Mouse	Active	[106]
	Paraguay	Dried leaf	MeOH ext	Croton oil-induced edema/**	Mouse	Active	[106]
	Paraguay	Dried leaf	H_2_O ext	Carrageenan-induced pedal edema/IP	Mouse	Active	[106]
	Paraguay	Dried leaf	MeOH ext	Carrageenan-induced pedal edema/IP	Mouse	Active	[106]
	Paraguay	Dried leaf	CH_2_Cl_2_ ext	Carrageenan-induced pedal edema/IP	Mouse	Active	[106]
Proteaceae							
*Lomatia hirsuta*	Chile	Dried leaf	Infusion	Carrageenan-induced pedal edema/Intragastric	Guinea pig	Active	[202]
Rhamnaceae							
*Trevoa trinervis*	Chile	Dried aerial parts	MeOH ext	Carrageenan-induced pedal edema/Intragastric	Guinea pig	Weak activity	[203]
	Chile	Dried aerial parts	Hexane ext	Carrageenan-induced pedal edema/Intragastric	Guinea pig	Weak activity	[203]
	Chile	Dried aerial parts	Dichloromethane ext	Carrageenan-induced pedal edema/Intragastric	Guinea pig	Weak activity	[203]
	Chile	Dried aerial parts	H_2_O ext	Carrageenan-induced pedal edema/Intragastric	Guinea pig	Weak activity	[203]
	Chile	Dried aerial parts	MeOH ext	Carrageenan-induced pedal edema/Intragastric	Guinea pig	Active	[203]
Rosaceae							
*Acaena splendens*	Chile	Dried bark + spines	CH_2_Cl_2_ ext	Carrageenan-induced pedal edema/Intragastric	Guinea pig	Weak activity	[204]
	Chile	Dried bark + spines	Infusion	Carrageenan-induced pedal edema/Intragastric	Guinea pig	Weak activity	[204]
	Chile	Dried bark + spines	MeOH ext	Carrageenan-induced pedal edema/Intragastric	Guinea pig	Weak activity	[204]
Kageneckia oblonga	Chile	Dried aerial parts	Hexane ext	Acetic acid-induced pedal edema/**	Mouse	Active	[205]
	Chile	Dried aerial parts	CHCl_3_-MeOH extract (2:1)	Acetic acid-induced pedal edema/**	Mouse	Active	[205]
	Chile	Dried aerial parts	MeOH ext	Acetic acid-induced pedal edema/**	Mouse	Active	[205]
	Chile	Dried aerial parts	CHCl_3_-MeOH extract (2:1)	Carrageenan-induced pedal edema/Intragastric	Guinea pig	Active	[205]
	Chile	Dried aerial parts	H_2_O soluble fraction	Carrageenan-induced pedal edema/Intragastric	Guinea pig	Active	[205]
	Chile	Dried aerial parts	MeOH ext	Carrageenan-induced pedal edema/Intragastric	Guinea pig	Active	[205]
	Chile	Dried aerial parts	Hexane ext	Carrageenan-induced pedal edema/Intragastric	Guinea pig	Active	[205]
Rubiaceae							
*Chiococca brachiata*	Brazil	Fresh root	Infusion	Dye diffusion assay/Intragastric	Mouse	active	[119]
*Coutarea hexandra*	Brazil	Dried stembark	EtOH (95%) ext	Carrageenan-induced pedal edema/Intragastric	Rat	Active	[206]
*Uncaria guianensis*	Peru	Dried bark	Lyophilized extract	**/**	Human adult	Active	[207]
*Uncaria tomentosa*	Peru	Freeze-dried bark	H_2_O ext	Carrageenan-induced pedal edema/Intragastric	Mouse	Active	[208]
	Peru	Freeze-dried bark	Hydro-alcoholic ext	Carrageenan-induced pedal edema/Intragastric	Mouse	Active	[208]
	Peru	Dried bark	Lyophilized extract	**/IP	Mouse	Active	[209]
	Peru	Dried bark	Lyophilized extract	**/Oral	Human adult	Active	[209]
	Peru	Dried bark	H_2_O ext	Cell Culture	In vitro	Active	[210]
	Peru	Dried bark	H_2_O ext	5-ht-induced pedal edema/Intragastric	Rat	Active	[211]
	Peru	Dried bark	Lyophilized extract	**/Oral	Human adult	Active	[207]
	Peru	Dried vine	Type ext not stated	**/Route not given	Human adult	Active	[212]
	Peru	Dried bark	Pet ether ext	5-ht-Induced pedal edema/IP	Rat	Active	[213]
	Peru	Dried bark	H_2_O ext	Chronic intestinal inflammation induced by indomethacin/**	Rat	Active	[210]
	Peru	Dried root	Rootbark	Convulsions strychnine-induced/carrageenan-induced pedal edemaI/Intragastric	Rat	Active	[214]
	Peru	Dried bark	EtoAc ext	5-ht-Induced pedal edema/Intragastric	Rat	Active	[211]
	Peru	Part not specified	Type ext not stated	**/**	Human adult	Equivocal	[215]
Rutaceae							
*Zanthoxylum chiloperone*	Brazil	dried leaf	Pet ether ext	Carrageenan-induced pedal edema/Intragastric		Active	[216]
Sapindaceae							
*Dodonaea viscosa*	Brazil	Dried leaf	EtOH (70%) ext	Carrageenan-induced pedal edema/Intragastric	Rat	Active	[217]
Sapotaceae							
*Bumelia sartorum*	Brazil	Dried rootbark	EtOH (95%) ext	Carrageenan-induced pedal edema/Gastric Intubation	Rat	Active	[218]
*Scoparia dulcis*	Brazil	Dried entire plant	EtOH (95%) ext	Histamine-induced edema/carrageenan-induced pedal edema/Intragastric	Rat	Active	[219]
	Brazil	Dried entire plant	H_2_O ext	Carrageenan-induced pedal edema/Intragastric	Rat	Inactive	[219]
	Brazil	Dried entire plant	EtOH (95%) ext	Dextran-induced pedal edema/Intragastric	Rat	Active	[219]
	Brazil	Dried entire plant	EtOH (95%) ext	Cotton pellet granuloma/Intragastric	Rat	Inactive	[219]
Simaroubaceae							
*Simaba cedron*	South America	Seed	Ether ext	**/SC	Rat	Inactive	[220]
	South America	Seed	Pet ether ext	**/SC	Rat	Inactive	[220]
	South America	Seed	EtOH (95%) ext	Cotton pellet granuloma/SC	Rat	Weak activity	[220]
	South America	Seed	H_2_O ext	Dextran-induced pedal edema/SC	Rat	Weak activity	[220]
Solanaceae							
*Brunfelsia bonodora*	Peru	Dried part not specified	EtOH (95%) ext	Carrageenan-induced pedal edema/IP	Rat	Active	[132]
*Brunfelsia uniflora*	Brazil	Root	MeOH ext	Carrageenin-induced pedal edema/Oral	Rat	Active	[221]
	Brazil	Root	CHCl_3_ ext	Data incomplete/Oral	Rat	Active	[222]
	Brazil	Fresh leaf	Infusion	Dye diffusion assay/Intragastric	Mouse	Inactive	[119]
*Solanum ligustrinum*	Chile	Dried aerial parts	Infusion	Carrageenan-induced pedal edema/Intragastric	Guinea pig	Weak activity	[223]
	Chile	Dried aerial parts	Decoction	Carrageenan-induced pedal edema/Intragastric	Guinea pig	Weak activity	[223]
	Chile	Dried aerial parts	MeOH ext	Carrageenan-induced pedal edema/Intragastric	Guinea pig	Weak activity	[223]
	Chile	Dried aerial parts	Dichloromethane ext	Carrageenan-induced pedal edema/Intragastric	Guinea pig	Weak activity	[223]
	Chile	Dried aerial parts	H_2_O ext	Carrageenan-induced pedal edema/Intragastric	Guinea pig	Weak activity	[223]
	Chile	Dried aerial parts	Pet ether ext	Carrageenan-induced pedal edema/Intragastric	Guinea pig	Weak activity	[223]
	Chile	Dried aerial parts	MeOH ext	Carrageenan-induced pedal edema/Intragastric	Guinea pig	Weak activity	[223]
*Solanum lycocarpum*	Brazil	Dried fruit	EtOH (95%) ext	Croton oil-induced edema/Intragastric	Mouse	Active	[224]
	Brazil	Dried fruit	Alkaloid fract	Carrageenan-induced pedal edema/SC	Mouse	Active	[224]
	Brazil	Dried fruit	Alkaloid fract	Croton oil-induced edema/SC	Mouse	Active	[224]
Turneraceae							
*Turnera ulmifolia*	Brazil	Dried entire plant	Hydro-alcoholic ext	Cotton pellet granuloma/Intragastric	Rat	Active	[225]
	Brazil	Dried entire plant	EtoAc ext	Carrageenan-induced pedal edema/Intragastric	Rat	Inactive	[225]
Verbenaceae							
*Bouchea fluminensis*	Brazil	Dried leaf	H_2_O ext	Carrageenan-induced pedal edema/Route not given	Rat	Active	[87]
	Brazil	dried aerial parts	EtOH (95%) ext	5-ht-induced pedal edema/Intragastric	Mouse	Active	[226]
	Brazil	dried aerial parts	EtOH (95%) ext	Histamine-induced edema/Intragastric	Mouse	Active	[226]
	Brazil	dried aerial parts	EtOH (95%) ext	Carrageenan-induced pedal edema/Intragastric	Mouse	Active	[226]
*Stachytarpheta cayennensis*	Brazil	Dried leaf	EtOH (70%) ext	Carrageenan-induced pedal edema/Intragastric	Rat	Weak activity	[227]
	Brazil	Dried leaf	Infusion	Carrageenan-induced pedal edema/Intragastric	Rat	Active	[227]
	Brazil	Dried entire plant	H_2_O ext	Dextran-induced pedal edema/carrageenan-induced pedal edema/histamine-induced edema/Intragastric	Mouse	Inactive	[227]
	Brazil	Dried leaf	Butanol ext	Carrageenan-induced pedal edema/IP	*Salvelinus alpinus*	Weak activity	[227]
	Brazil	Dried leaf	Butanol ext	Carrageenan-induced pedal edema/IP	Rat	Active	[228]
Winteraceae							
*Drimys winter*	Brazil	Dried bark	Hydro-alcoholic ext	PgE_2_ induced paw oedma/Intragastric	Rat	Equivocal	[229]
	Brazil	Dried bark	Hydro-alcoholic ext	Histamine-induced edema/Intragastric	Rat	Inactive	[229]
	Brazil	Dried bark	Hydro-alcoholic ext	Carrageenan-induced pedal edema/Intragastric	Rat	Active	[229]
	Brazil	Dried bark	Hydro-alcoholic ext	Dextran-induced pedal edema/Intragastric	Rat	Active	[229]
	Brazil	Dried bark	Hydro-alcoholic ext	Bradykinin-induced pedal edema/Intragastric	Rat	Weak activity	[229]
	Brazil	Dried bark	Hydro-alcoholic ext	Paw oedema/Intragastric	Rat	Weak activity	[229]
	Brazil	Dried bark	Hydro-alcoholic ext	Paf-acether induced paw oedema/Intragastric	Rat	Weak activity	[229]
	Brazil	Dried bark	Hydro-alcoholic ext	Ovalbumine induced paw oedema/Intragastric	Rat	Weak activity	[229]
Zingiberaceae							[229]
*Zingiber officinale*	Brazil	Fresh rhizome	Hydro-alcoholic ext	Carrageenan-induced pedal edema/IP	Rat	Active	[230]
	Brazil	Fresh rhizome	Hydro-alcoholic ext	5-ht-induced pedal edema/IP	Rat	Active	[230]
	Brazil	Fresh rhizome	Hydro-alcoholic ext	48180 compound-induced edema/**	Rat	Active	[230]
	Brazil	Fresh rhizome	Hydro-alcoholic ext	48180 and 5-ht induced skin edema/IP	Rat	Active	[230]
Zygophyllaceae							
*Larrea divaricata*	Argentina	Dried leaf	MeOH ext	Cotton pellet granuloma/Intragastric	Rat	Active	[231]
	Argentina	Dried leaf	H_2_O ext	Peritoneal macrophages/IP	Mouse	Active	[232]

** Incompleted dates; IP = intraperitoneal; SC = subcutaneous; EtOH = ethanolic extract ; H_2_O ext = aqueous extract; MeOH ext = methanol extract; EtoAc ext = ethyl acetate extract; CH_2_Cl_2_ ext = dichloromethane extract; CHCl_3_ ext = chloroformic extract ; CCl_4_ = chloroform; MeCl_2_ ext = dichloromethane extract; EtOH-H_2_O = crude aqueous/alcoholic extract; CHCl_3_-MeOH extract = dichloromethane and methanol extract.
